# Lifestyle Clusters of Diet Quality, Sleep, and Screen Time and Associations with Weight Status in Children from Madrid City: ENPIMAD Study

**DOI:** 10.3390/nu16132096

**Published:** 2024-06-30

**Authors:** África Peral-Suárez, Laura M. Bermejo, María Dolores Salas-González, Esther Cuadrado-Soto, María Del Carmen Lozano-Estevan, Viviana Loria-Kohen, Liliana G. González-Rodríguez, Aránzazu Aparicio, José Manuel Díaz-Olalla, Ana M. López-Sobaler

**Affiliations:** 1 Department of Nutrition and Food Science, Complutense University of Madrid, 28040 Madrid, Spain; africper@ucm.es (Á.P.-S.); mlbermej@ucm.es (L.M.B.); masala06@ucm.es (M.D.S.-G.); esther.cuadrado@ucm.es (E.C.-S.); mlozan16@ucm.es (M.D.C.L.-E.); vloria@ucm.es (V.L.-K.); liligonz@ucm.es (L.G.G.-R.); araparic@ucm.es (A.A.); 2 VALORNUT Research Group, Department of Nutrition and Food Science, Faculty of Pharmacy, Complutense University of Madrid, 28040 Madrid, Spain; 3 San Carlos Health Research Institute (IdISSC), 28040 Madrid, Spain; 4 Madrid Salud, Ayuntamiento de Madrid, 28007 Madrid, Spain; manueldiazolalla@yahoo.es

**Keywords:** lifestyle, clusters, diet, sleep, sedentary, children, socio-economic status, food insecurity, weight status, abdominal obesity

## Abstract

Background: Childhood overweight and obesity is a global concern and has increased in Spain over the last decades. Combinations of lifestyle behaviors (i.e., diet, sleep, and sedentarism) are highly related to weight status. Therefore, this study aimed to identify lifestyle patterns among children from Madrid City, and analyze associations with the prevalence of overweight, obesity, and abdominal obesity, considering socio-economic factors. Methods: A cross-sectional analysis was conducted on 4545 children from the ENPIMAD study with data on diet, sleep, anthropometric, and socio-economic variables. K-means cluster analysis was used to identify lifestyle clusters, and logistic regressions were used to examine the associations between socio-economic indicators and cluster membership, and between clusters and weight status. Results: Findings show three lifestyle clusters (healthy, mixed, and unhealthy), with boys and older children more represented in the unhealthy cluster. Food insecurity and low socio-economic status were associated with unhealthier clusters in boys and girls. Children in unhealthier clusters were more likely to have obesity and abdominal obesity. However, these associations disappeared in girls after controlling for food insecurity. Conclusion: These results provide insight into the combination of behaviors and socio-economic factors associated with childhood obesity that may aid in the design of future interventions.

## 1. Introduction

Overweight and obesity during childhood have been associated with a range of psychosocial and physiological conditions, such as low self-esteem, depression, altered pubertal development and increased cardio-metabolic risk in adulthood [1,2]. Furthermore, abdominal adiposity is positively linked to increased blood pressure and dyslipidemia in children, independent of body mass index (BMI) [3,4].

According to the World Health Organization (WHO), 390 million children and adolescents aged 5–19 years had excess weight in 2022, representing a prevalence of 20% [5]. Over the past two decades, the prevalence of excess of body weight has increased by more than 10% among children aged between 7 and 13 years and by more than 15% among children aged between 2 and 6 years in Spain, with the prevalence of overweight in 2021 being 15% and 35%, respectively [6]. Overweight and obesity prevalence also seems to vary among different regions within the country, being lower in the northern regions, and showing differences in the distribution between sexes [7,8].

Disentangling the key determinants of the development of childhood overweight and obesity is essential for designing effective interventions. In this context, although excess weight has a complex multifactorial etiology, there are some modifiable lifestyle behaviors that are highly correlated with this condition [1,2]. These obesogenic lifestyle behaviors include unhealthy dietary habits (e.g., low fruit and vegetable consumption, high fast-food, sweets, or snacks consumption, etc.), insufficient sleep, low physical activity, and high levels of sedentary behavior [1,9]. Different studies have linked these behaviors independently with the development of overweight and obesity [9]. However, recent investigations show that unhealthy lifestyle behaviors tend to co-occur within groups of children, and they have synergistic effects on health outcomes, making the combination of some unhealthy behaviors more harmful than the effect of each individual one [10,11]. In this sense, although several studies have examined relationships between individual sleep indicators (e.g., sleep duration, sleep quality) and childhood overweight and obesity [12,13,14], very few studies have examined co-occurring patterns of sleep duration together with other obesogenic behaviors (e.g., screen time, diet) in relation to health outcomes [15].

Previous studies have described that lifestyle cluster membership may be associated with different factors [11]. For example, girls tend to be more represented in patterns characterized by low physical activity and high sedentary behavior but healthy diets, while boys tend to be more represented in patterns characterized by high physical activity and similar sedentary behavior but unhealthier diets [15]. Additionally, older children and those from low-status socio-economic backgrounds tend to be more represented in unhealthy patterns combining three or four unfavorable behaviors [11,16]. However, there are socio-economic indicators, such as household food insecurity, which have previously been related to overweight, obesity, and associated comorbidities in children [17,18]. In fact, food insecurity has been related to different lifestyle behaviors implied in excess weight independently (i.e., poorer sleep or diet quality) [19,20], but how it interacts with lifestyle patterns is still unknown. Thus, a better understanding of the factors associated with lifestyle clusters is required to underpin public health interventions designed to foster positive changes in health behaviors from childhood [15].

Therefore, the aims of this study were (i) to identify patterns of lifestyle behaviors among children from Madrid City, (ii) to analyze associations between socio-economic indicators and the lifestyle patterns of children, and (iii) to analyze associations between behavioral patterns of children and the prevalence of overweight, obesity, and abdominal obesity.

## 2. Materials and Methods

### 2.1. Study Design and Sample

The data were obtained from the ENPIMAD study, an observational cross-sectional study conducted in 2015 among a representative sample of children aged 3 to 12 years residing in Madrid (Spain) and enrolled in public and private kindergarten and primary school centers in the city.

**Figure 1:**
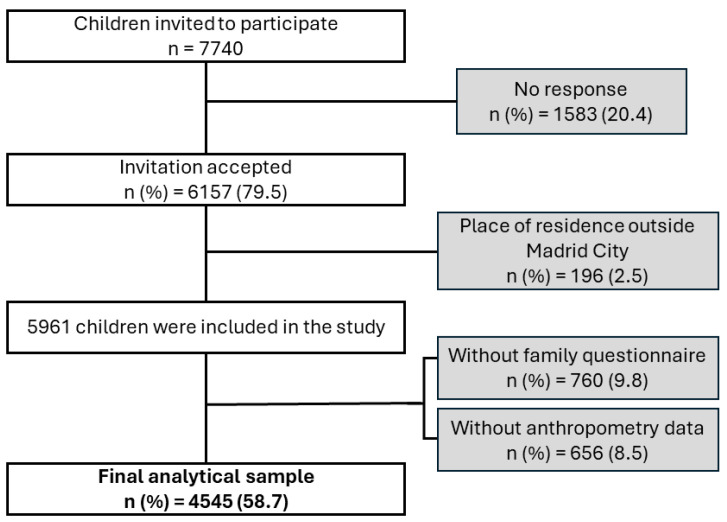
Sample selection flowchart.

A multi-stage cluster sampling technique with stratification of the first-stage units was used for sample selection. To achieve a minimum sample of 5500 children, the schools (first stage) were stratified into four strata (high development, mid-high development, mid-low development, and low development) based on the socio-economic development of the district they belonged to. This classification was made according to three indicators of socio-economic development: (i) life expectancy at birth, (ii) percentage of residents aged 30 to 64 years with education level above secondary school degree, and (iii) gross income per capita. The schools’ selection was carried out by sampling with probability proportional to size (measured in terms of the estimated number of kindergarten and primary school students per center). A total of 84 centers were contacted, from which a valid sample of 60 centers (71.4%) was obtained (27 public schools and 33 private schools). Between 5 and 6 classrooms per school (with only one classroom per course between first year of kindergarten and sixth year of primary school) were randomly selected (second stage), obtaining a sample of 336 classrooms. The 7740 children enrolled in the selected classrooms (third stage) received an invitation to participate in the study. Finally, 4545 children accepted the invitation to participate, provided the questionnaire completed by their parents/carers, and attended the anthropometric assessment (Figure 1).

All procedures were carried out in accordance with the Declaration of Helsinki. Parents and participants provided written informed consent prior to completing all assessments.

### 2.2. Measures

Parents/carers of children completed a family questionnaire including data on lifestyle behaviors of their children (diet quality, sleep, and screen time habits) and socio-economic data. Furthermore, a visit to the schools was made to collect anthropometric data.

#### 2.2.1. Lifestyle Behaviors: Diet, Sleep, and Screen Time

Diet quality was evaluated using the KIDMED questionnaire [21], which is a validated questionnaire that measures adherence to the Mediterranean Diet (using a scale from 0 to 12). This questionnaire was included in the family questionnaire completed by the parents/carers. A higher KIDMED score indicates greater adherence to the Mediterranean Diet. Furthermore, children were classified based on their KIDMED score as exhibiting low (0–3), moderate (4–7), or high (8–12) adherence to the Mediterranean Diet.

Sleep time was reported as an open question asking the time spent sleeping (including naps) on weekdays and weekends. For the present analyses, the mean daily time spent sleeping was calculated using the formula: (time spent on weekdays in hours × 5 + time spent on weekends in hours × 2)/7.

Time spent (in hours) watching TV and playing electronic games on weekdays and weekends was reported separately. In both cases, closed questions were used including the following answer options: ‘Never’, ‘less than 1 h/day’, ‘1 h/day’, ‘2 h/day’, ‘3 h/day’, ‘4 h/day’, and ‘5 or more h/day’. For the present analyses, the mean daily time spent watching TV and playing electronic games was calculated using the formula: (time spent on weekdays in hours × 5 + time spent on weekends in hours × 2)/7. Subsequently, the results were reintegrated into a single screen-time variable by summing daily time spent watching TV and daily time spent playing electronic games.

#### 2.2.2. Socio-Economic Variables

The family questionnaire also included two scales validated for the Spanish population: the Household Food Insecurity Access Scale (HFIAS) 2007 [22] and the Family Affluence Scale (FAS) version III [23]. The HFIAS scale identifies and distinguishes the severity of food insecurity (food access component) in households in different cultural contexts. For the present analyses, the HFIAS scale was dichotomized as ‘with food insecurity’ and ‘without food insecurity’. The FAS scale classifies the socio-economic status (SES) of children’s families as ‘low’, ‘mid’, or ‘high’.

#### 2.2.3. Anthropometric Data

Data on weight, height, and waist circumference (WC) were collected by trained researchers during a school visit using standardized methods. Children were asked to be barefoot and wearing light clothes during the measuring process. Weight was assessed using calibrated Tanita UM-076 scales (Tanita UK, Yiewsley, Middlesex, UK), height was assessed using a portable stadiometer model MZ 10042 (WTEC, Madrid, Spain), and waist circumference was measured following the WHO criteria [24]. Body Mass Index (BMI) was calculated as weight (kg)/(height (m))^2^, and participant’s weight status was categorized as ‘not overweight, including underweight’, ‘overweight’, and ‘obese’, based on the WHO BMI for age z-score cut-points of +1.0 and +2.0 [25,26]. Waist-to-height ratio (WtHR) was calculated as WC (cm)/height (cm), and abdominal obesity was defined as WtHR ≥ 90th percentile until six years of age [27] and WtHR ≥ 0.5 from six years and above [28].

### 2.3. Statistical Analysis

Sample weight factors for each participant were calculated to account for nonresponses and to weight the sample to known population demographic characteristics. Analyses were conducted using IBM SPSS Statistics software version 27.0 (IBM Corp., Armonk, NY, USA). Sample characteristics were computed by sex and summarized as median inter-quartile range (IQR) for continuous variables, and frequencies and percentages for categorical variables. A Kolmogorov–Smirnov test and histograms were used to assess the variable distribution. For comparing sample characteristics at baseline between sexes, independent Mann–Whitney U tests were used for differences in continuous non-parametric variables, and chi-square tests were used for differences in proportions.

A two-step clustering procedure was performed, based on KIDMED score, sleep duration, and screen time. As the variables used had different arithmetic scales, z-scores were calculated to standardize the dataset before clustering, to avoid a greater contribution to the distance of variables having larger ranges than variables with smaller ranges. First, a dendrogram was generated through agglomerative hierarchical clustering using Ward’s method to identify the possible cluster solutions, from which a three-cluster and a four-cluster solution emerged. To ensure the correct pre-selection of the number of clusters, we have additionally calculated with the elbow method to support this estimation and find the best solution, obtaining the same result. Subsequently, non-hierarchical, k-means partitioning cluster analysis was performed to create the three clusters and four clusters. To study the reliability of each cluster solution, in order to select the most optimal, the number of iterations needed for each solution to stabilize was analyzed. The three-cluster solution stabilized in 10 iterations, while the four-cluster solution took more than 15 iterations. Therefore, the three-cluster solution was used for the present analyses. Kruskal–Wallis tests (for continuous variables) followed by Bonferroni post hoc comparisons and chi-square tests (for categorical variables) were used to assess the group differences.

Multinomial logistic regressions and the calculation of the corresponding odds ratios (OR) were used to examine the associations between socio-economic variables and lifestyle clusters, as well as between lifestyle clusters and weight status. Binary logistic regressions and the calculation of the corresponding odds ratios (OR) were used to examine the associations between clusters and abdominal obesity. A *p*-value lower than 0.05 was considered significant for all tests.

## 3. Results

The final analytical sample resulted in 4545 children (48.7% girls and 51.3% boys) representing the city of Madrid whose average age was 7.3 years (SD = 2.8).

### 3.1. Sample Characteristics by Sex

Sample characteristics are described in Table 1. Of the total sample, 45.9% had a low socio-economic status, with no differences among sexes. Regarding body composition, 40.9% had excess weight, with obesity being higher in boys than in girls (18.9% vs. 12.3%; *p* < 0.001). Most of the students (91.5%) showed moderate to high adherence to the Mediterranean Diet, and the average screen time duration was 2.1 (1.4–3.1) h. However, boys exhibited a greater proportion of low adherence to the Mediterranean Diet (9.6% vs. 7.3%; *p* = 0.005) and higher screen time (median (IQR) = 2.3 (1.6–3.3) h/day vs. 2.0 (1.3–2.9) h/day; *p* < 0.001) compared to girls.

### 3.2. Characteristics of Lifestyle Clusters

**Figure 2:**
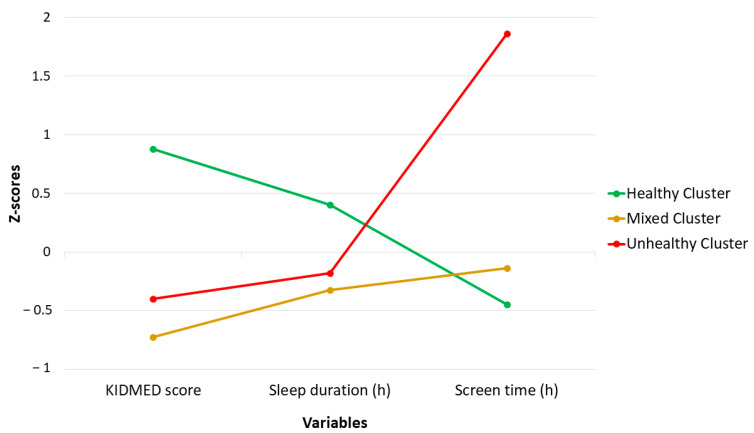
Lifestyle clusters.

Three lifestyle cluster solutions were produced: Cluster 1 was classified as Healthy Cluster (HC), Cluster 2 was classified as Mixed Cluster (MC), and Cluster 3 was classified as Unhealthy Cluster (UC) (Figure 2). Of the sample, 42.2% was in the HC, 42.8% was in the MC, and 15.0% was in the UC.

Table 2 shows the characteristics of the children comprised by the different clusters. In the HC, there was a similar proportion of girls and boys, while the proportion of boys increased in unhealthier clusters, and children in the UC were older than in the HC and UC. The UC was characterized by a higher percentage of children with low school-district development, food insecurity, and low SES compared to the other two clusters. Similarly, the UC included a higher percentage of children with excess weight (overweight and obesity) and abdominal obesity than the other two clusters.

Children in the HC had higher KIDMED scores, slept longer, and had lower screen time than children in MC and UC. Sleep duration was similar in the MC and UC. Children in the MC had the lowest mean KIDMED score, and children in the UC had the highest screen time (Table 2).

### 3.3. Socio-Economic Factors Associated with Cluster Membership

After controlling for age and the other socio-economic factors (Model 2), boys from low and mid-low school districts were more than twice as likely to be in the UC than those from high-development school districts. Boys with food insecurity were more likely to be in the UC than those without food insecurity, and boys from a low SES were almost twice as likely to be in the MC and four times more likely to be in the UC than those from a high SES (Table 3).

Among girls, after controlling for age and the other socio-economic factors (Model 2), those with food insecurity were more likely to be in the MC and UC than those without food insecurity. Girls from a low-class SES were 2.5 times more likely to be in the MC and 5 times more likely to be in the UC than those from a high-class socio-economic background. Also, girls from a mid-class SES were more likely to be in the MC and the UC than girls from a high-class SES (Table 3).

### 3.4. Likelihood of Overweight and Obesity Depending on Cluster Membership

Associations between cluster membership and prevalence of overweight, obesity, and abdominal obesity are shown in Table 4. Among boys, after controlling for age and SES (Model 2) or age and food insecurity (Model 3), those who were in the MC were more likely to be overweight than those in the HC. Boys in the UC were more likely to have obesity or abdominal obesity than boys in the HC. These associations remained after controlling for food insecurity and SES together (Model 4).

Among girls, after controlling for age and socio-economic status (Model 2) or age and food insecurity (Model 3), those in the UC were more likely to have obesity and abdominal obesity. However, these associations disappeared after controlling for food insecurity and SES together (Model 4). However, OR for obesity of girls in the UC was 1.47, showing a marginal significance (*p* = 0.056).

## 4. Discussion

This study aimed to identify lifestyle patterns among children from Madrid City, as well as to analyze associations between these patterns and the prevalence of overweight, obesity, and abdominal obesity, taking into account socio-economic factors associated with cluster membership. Results showed three lifestyle clusters involving adherence to Mediterranean Diet, sleep, and screen time, with girls and younger children being more represented in the healthier cluster. Food insecurity and low SES were associated with unhealthier clusters in both boys and girls. Children in the unhealthier cluster were more likely to have obesity and abdominal obesity. However, in girls these associations disappeared after controlling for both food insecurity and SES.

The present study identified three lifestyle clusters combining diet quality, sleep duration, and screen time: a HC, a MC, and an UC. In line with these results, unhealthy clusters combining lower adherence to Mediterranean Diet, lower sleep duration, and higher screen time have been previously described in the Spanish population aged 7–17 years [29]. Analyzing characteristics of children belonging to each cluster, older children were more represented in the unhealthier cluster. In the present results, the UC is characterized by significantly higher screen time than the other clusters, and previous studies have shown an increase in screen time and sedentary behavior from childhood to adolescence [30,31,32]. This fact, combined with the existing evidence that high screen time is associated with other poor lifestyle habits, such as poorer diet quality [33,34], supports our findings of poorer lifestyles in older children. Furthermore, as this sample includes children aged 3 to 12 years, older children are transitioning into adolescence, which is a period strongly associated with a decline in healthy lifestyle behaviors [35,36] due to a higher exposure to social media, advertisements for unhealthy foods, and increased autonomy around decisions related to lifestyle [36,37]. In the present study, boys were more represented in unhealthier clusters, which contrasts with previous studies in which girls showed unhealthier lifestyle patterns than boys [38]. However, previous systematic reviews highlight that most of the children had mixed lifestyle patterns, with boys more represented in clusters including high physical activity and poor diet quality, while girls were more represented in clusters including high sleep duration and diet quality [15,39], as observed in the present study.

Given the relevance of identifying the factors that determine children’s lifestyles for designing interventions to prevent or reduce overweight and obesity, the present study focused on which socio-economic indicators (school district, food insecurity, and SES) were related to the observed lifestyle patterns.

Low and mid-low school district development were associated with the UC in boys independently of other socio-economic factors, while this association was not significant in girls when controlling for other socio-economic factors. Previous studies have shown poorer dietary behaviors and lower levels of physical activity among children living in more economically disadvantaged neighborhoods [40]. Furthermore, some characteristics of high-development neighborhoods, such as green spaces or schools with sports facilities and their own kitchens, have a positive influence on children’s lifestyles [8,41,42]. In this sense, sex differences have also been described. For example, Sanders et al. [41] found associations between the availability of green spaces in neighborhoods with a significant increase of physical activity and decrease of screen time in boys, but not in girls.

In the present study, children with food insecurity were more likely to be in unhealthier clusters (MC and UC). Although there is a lack of evidence examining the influence of household food insecurity on lifestyle clusters that combine different behaviors, poorer dietary patterns characterized by low fruit and vegetable intake and high snacking, as well as longer screen time, have been described in children from households with high food insecurity [43,44].

Also, the present results showed that children in the low SES level (classified using FAS scale) were more likely to be in the MC and UC, which was also observed in girls in the mid SES level. There are also insufficient studies that examine the association of lifestyle clusters and SES levels assessed using the FAS scale. Consequently, making comparisons is challenging. However, some studies have analyzed the relationship between SES level measured using FAS scale and individual lifestyle behaviors, finding low SES strongly associated with poorer lifestyle. For example, Yannakoulia et al. [45] found that children (aged 3 to 12 years) in the medium- or high-SES groups showed higher KIDMED scores compared with those in the low-SES group, and Borracino et al. [46] found associations between higher-SES groups and higher rates of moderate-to-vigorous physical activity in adolescents.

It is important to highlight that, in terms of lifestyle clusters, previous studies have shown associations between other indicators of SES (i.e., parental education, household income, etc.) and unhealthier combinations of lifestyle behaviors mainly characterized by high screen time and inadequate dietary habits [47,48].

The associations between the described lifestyle clusters and the prevalence of overweight, obesity, and abdominal obesity have also been analyzed in the present study. In the results, being in the UC was associated with a higher likelihood of obesity and abdominal obesity in both boys and girls, consistent with previous studies in Spanish children and adolescents [49]. However, sex differences were found when controlling for various socio-economic indicators. In boys, the associations remained after adjusting for SES (FAS) and food insecurity (HFIAS), suggesting that the UC confers an increased likelihood for obesity and abdominal obesity in boys, regardless of their socio-economic background. In fact, longitudinal studies have shown that children with lifestyle patterns characterized by low diet quality, high sedentary behavior, and inactivity were more likely to develop overweight and obesity, as well as increase their body fat over time [50,51]. On the other hand, after adjusting for SES (FAS) and food insecurity (HFIAS), the associations between lifestyle clusters and excess weight and abdominal obesity were no longer significant in girls. Previous studies conducted on children have already shown disparities between boys and girls in the relationship between household food insecurity and obesity. For example, Bae and Choi [52] found that girls with food insecurity were more likely to be obese, while boys with food insecurity were less likely to be obese than those without food insecurity. These results suggest potential sex differences in how lifestyle and socio-economic background interact in the development of overweight and obesity in children, highlighting the need for further studies to examine the etiology of weight excess by separating boys and girls and considering lifestyle patterns and different socio-economic indicators.

Therefore, future interventions to prevent childhood overweight and obesity should consider targeting multiple lifestyle behaviors simultaneously, and be designed according to the sex, age, and socio-economic status of the target population to increase their effectiveness.

### Strengths and Limitations

To the best of our knowledge, this is the first study to examine the associations between clusters of multiple lifestyle behaviors in children and different indicators of socio-economic status, measured using validated scales. Strengths of the study include the carefully designed methodology used in the ENPIMAD study, conducted among a representative sample of the population aged 3–12 years from Madrid City, the inclusion of multiple lifestyle behaviors, and thorough analysis of clustering. However, conclusions must be drawn in light of the limitations. The cross-sectional design only allows for the observation of associations. Thus, interventions and longitudinal studies are needed to make causal inferences from the observed associations [53]. However, this design allows us to describe the prevalence of the excess weight and associated factors in Madrid City, suggesting hypotheses for future more in-depth studies [54]. Although validated questionnaires were used to assess diet quality (KIDMED), SES (FAS), and food insecurity (HFIAS), the cluster analysis variables (dietary variables, sleep duration, and screen time) and sociodemographic variables were all parent-reported, which could result in measurement error due to socially desirable responses and/or inaccurate recall [55,56,57]. As our sample is representative of the city of Madrid, the results are not generalizable to other regions in the country. This study employed a two-step clustering approach utilizing Ward’s hierarchical clustering and the elbow method to identify initial cluster centers, followed by k-means for final cluster assignment. This approach balanced the need for simplicity and efficiency in analyzing a dataset while achieving a meaningful group structure based on observable variables. However, k-means clustering might not fully capture the underlying complexity due to potential limitations in accounting for unobserved heterogeneity and non-linear relationships between variables. Future studies exploring methods like Latent Profile Analysis (LPA) could address these limitations and potentially reveal a more nuanced understanding of the group structure. Finally, due to limitations in the questionnaire in terms of questions about physical activity, it was not possible to obtain data on overall moderate-to-vigorous physical activity. Therefore, future research analyzing the relationship between lifestyle patterns, socio-economic factors, and weight status should incorporate physical activity indicators.

## 5. Conclusions

A HC, a MC, and an UC combining diet quality, sleep duration, and screen time were identified among children from Madrid City. Low SES and food insecurity have shown associations with UC membership. Children in the UC were more likely to have obesity and abdominal obesity regardless of their SES, but in girls these associations may be mediated by food insecurity. Future research including physical activity as a factor in the analysis of lifestyle in children is needed. Interventions to prevent childhood overweight and obesity should consider targeting multiple lifestyle behaviors simultaneously, and be designed according to the sex, age, and socio-economic status of the target population.

## Figures and Tables

**Table 1:** Sample characteristics by sex.

		Total	Boys	Girls	*p*
**n**	n (%)	4545	2331 (51.3)	2214 (48.7)	
**Age (years)**	median (IQR)	7.0 (5.0–10.0)	7.0 (5.0–10.0)	7.0 (5.0–10.0)	0.718
**School district**					0.876
Low	n (%)	1203 (26.5)	619 (26.5)	584 (26.4)	
Mid-low	n (%)	1562 (34.4)	793 (34.0)	769 (34.8)	
Mid-high	n (%)	1090 (24.0)	571 (24.5)	519 (23.4)	
High	n (%)	690 (15.2)	349 (15.0)	342 (15.4)	
**Food insecurity ^¥^**					0.076
With food insecurity	n (%)	812 (18.1)	414 (17.9)	398 (18.2)	
Without food insecurity	n (%)	3683 (81.9)	1897 (82.1)	1785 (81.8)	
**Socio-economic status ^¥^**					0.922
Low class	n (%)	2085 (45.9)	1062 (45.6)	1023 (46.2)	
Mid class	n (%)	1852 (40.8)	952 (40.8)	901 (40.7)	
High class	n (%)	568 (12.5)	296 (12.7)	272 (12.3)	
Undetermined	n (%)	40 (0.9)	22 (0.9)	18 (0.8)	
**Weight status**					**<0.001**
Underweight	n (%)	44 (1.0)	24 (1.0)	19 (0.9)	
Normal weight	n (%)	2636 (58.1)	1277 (54.8)	1359 (61.5)	
Overweight	n (%)	1150 (25.3)	588 (25.3)	561 (25.4)	
Obesity	n (%)	710 (15.6)	439 (18.9)	271 (12.3)	
**Abdominal obesity**	n (%)	1411 (31.1)	724 (31.2)	687 (31.1)	0.962
**Adherence to MD**					**0.005**
Low adherence	n (%)	385 (8.5)	225 (9.6)	161 (7.3)	
Medium adherence	n (%)	3199 (70.4)	1640 (70.4)	1559 (70.4)	
High adherence	n (%)	960 (21.1)	466 (20.0)	494 (22.3)	
**Sleep duration (h/day)**	median (IQR)	9.8 (9.5–10.5)	9.8 (9.5–10.5)	9.8 (9.5–10.5)	0.777
**Screen time (h/day)**	median (IQR)	2.1 (1.4–3.1)	2.3 (1.6–3.3)	2.0 (1.3–2.9)	**<0.001**

(a) MD: Mediterranean Diet, IQR: inter-quartile range. ^¥^ Food insecurity was classified according to the HFIAS scale, and socio-economic status was classified according to the FAS scale. (b) Mann–Whitney U tests were used for continuous variables and chi-square tests were used for categorical variables. Significant differences (*p* < 0.05) are marked as bold.

**Table 2:** Characteristics of the sample from different clusters.

		Healthy Cluster (HC)	Mixed Cluster (MC)	Unhealthy Cluster (UC)	*p*
**Sex**					**<0.001**
Boys	n (%)	952 (49.6)	996 (51.3)	383 (56.1)	
Girls	n (%)	967 (50.4)	948 (48.7)	300 (43.9)	
**Age (years)**	Median (IQR)	7.0 (5.0–9.0) ^a^	7.0 (5.0–10.0) ^b^	8.0 (6.0–10.0) ^c^	**<0.001**
**School district**					**<0.001**
Low	n (%)	412 (21.5)	555 (28.6)	236 (34.5)	
Mid-low	n (%)	623 (32.5)	646 (33.2)	293 (43.0)	
Mid-high	n (%)	546 (28.5)	454 (23.3)	90 (13.2)	
High	n (%)	337 (17.6)	289 (14.9)	64 (9.3)	
**Food insecurity ^¥^**					**<0.001**
With food insecurity	n (%)	229 (12.0)	373 (19.5)	210 (31.3)	
Without food insecurity	n (%)	1677 (88.0)	1545 (80.5)	461 (68.7)	
**Socio-economic status ^¥^**					**<0.001**
Low class	n (%)	656 (34.2)	963 (49.5)	466 (68.2)	
Mid class	n (%)	908 (47.3)	771 (39.6)	174 (25.5)	
High class	n (%)	341 (17.8)	193 (10.0)	34 (4.9)	
Undetermined	n (%)	14 (0.7)	17 (0.9)	9 (1.3)	
**Weight status**					**<0.001**
Underweight	n (%)	16 (0.8)	14 (0.7)	14 (2.1)	
Normal weight	n (%)	1224 (63.8)	1086 (55.9)	327 (48.0)	
Overweight	n (%)	434 (22.7)	535 (27.6)	180 (26.5)	
Obesity	n (%)	243 (12.7)	307 (15.8)	160 (23.5)	
**Abdominal obesity**	n (%)	542 (28.3)	603 (31.1)	266 (39.2)	**<0.001**
**KIDMED score**	Median (IQR)	7.0 (7.0–8.0) ^a^	5.0 (4.0–5.0) ^b^	5.0 (4.0–6.0) ^c^	**<0.001**
**Adherence to MD**					**<0.001**
Low adherence	n (%)	0 (0.0)	298 (15.3)	88 (12.8)	
Medium adherence	n (%)	999 (52.0)	1646 (84.7)	555 (81.3)	
High adherence	n (%)	920 (48.0)	0 (0.0)	40 (5.9)	
**Sleep duration (h/day)**	Median (IQR)	10.2 (9.8–10.7) ^a^	9.6 (9.1–10.1) ^b^	9.5 (8.8–10.8) ^b^	**<0.001**
**Screen time (h/day)**	Median (IQR)	1.6 (1.1–2.3) ^a^	2.2 (1.6–2.9) ^b^	4.9 (4.3–5.7) ^c^	**<0.001**

(a) MD: Mediterranean Diet, IQR: inter-quartile range. ^¥^ Food insecurity was classified according to the HFIAS scale, and socio-economic status was classified according to the FAS scale. (b) Kruskal–Wallis tests were used for continuous variables and chi-square tests were used for categorical variables. Significant differences (*p* < 0.05) are marked as bold. Pairwise differences in continuous variables were obtained using Bonferroni post hoc comparisons and marked with superscripts (^a–c^). When these subscripts are different, there are significant differences between pairs. (c) Bonferroni post hoc *p*-values for pairwise differences: Age (HC vs. MC: *p* = 0.008); Sleep duration (MC vs. UC: *p* = 0.572); all other pairwise differences *p* < 0.001.

**Table 3:** Odds ratios and 95% confidence intervals for cluster membership depending on socio-economic factors by sex.

			Boys				Girls			
			Mixed Cluster		Unhealthy Cluster		Mixed Cluster		Unhealthy Cluster	
			OR (95% CI)	*p*	OR (95% CI)	*p*	OR (95% CI)	*p*	OR (95% CI)	*p*
Model 1: Unadjusted	School district development	Low	1.66 (1.25, 2.20)	**<0.001**	4.43 (2.80, 6.99)	**<0.001**	1.50 (1.13, 2.00)	**0.006**	2.04 (1.33, 3.15)	**0.001**
		Mid-low	1.16 (0.89, 1.52)	0.275	3.42 (2.19, 5.32)	**<0.001**	1.26 (0.96, 1.66)	0.095	1.80 (1.19, 2.73)	**0.005**
		Mid-high	1.01 (0.76, 1.33)	0.949	1.00 (0.60, 1.67)	0.993	0.93 (0.69, 1.24)	0.605	0.77 (0.48, 1.24)	0.288
	Food insecurity (HFIAS scale)	With insecurity	1.63 (1.26, 2.10)	**<0.001**	3.60 (2.70, 4.81)	**<0.001**	1.92 (1.50, 2.47)	**<0.001**	3.03 (2.20, 4.16)	**<0.001**
	SES (FAS scale)	Low class	2.26 (1.71, 2.99)	**<0.001**	7.10 (4.32, 11.65)	**<0.001**	3.02 (2.25, 4.06)	**<0.001**	7.30 (4.13, 12.90)	**<0.001**
		Mid class	1.29 (0.98, 1.70)	0.067	1.73 (1.03, 2.90)	**0.039**	1.76 (1.31, 2.37)	**<0.001**	2.22 (1.23, 4.00)	**0.008**
Model 2: Adjusted	School district development	Low	1.30 (0.97, 1.75)	0.092	2.65 (1.65, 4.25)	**<0.001**	1.18 (0.87, 1.59)	0.280	1.23 (0.78, 1.94)	0.366
		Mid-low	0.98 (0.75, 1.30)	0.910	2.33 (1.48, 3.69)	**<0.001**	1.08 (0.81, 1.43)	0.607	1.35 (0.88, 2.08)	0.172
		Mid-high	0.99 (0.75, 1.32)	0.967	0.94 (0.56, 1.59)	0.827	0.92 (0.69, 1.24)	0.605	0.62 (0.37, 1.02)	0.060
	Food insecurity (HFIAS scale)	With insecurity	1.17 (0.88, 1.55)	0.278	1.71 (1.24, 2.35)	**<0.001**	1.41 (1.01, 1.88)	**0.007**	1.81 (1.29, 2.54)	**<0.001**
	SES (FAS scale)	Low class	1.99 (1.47, 2.69)	**<0.001**	3.95 (2.34, 6.68)	**<0.001**	2.57 (1.87, 3.51)	**<0.001**	5.05 (2.80, 9.10)	**<0.001**
		Mid class	1.23 (0.93, 1.63)	0.146	1.40 (0.82, 2.36)	0.214	1.68 (1.24, 2.27)	**<0.001**	1.95 (1.07, 3.54)	**0.028**

(a) CI: Confidence interval; FAS: Family Affluence Scale; HFIAS: Household Food Insecurity Access Scale; OR: Odds-ratio; SES: socio-economic status. (b) Reference categories for independent variables: School district development = high; Food insecurity = without insecurity; SES = high class. (c) Model 2 is adjusted by age and other socio-economic factors. (d) Multinomial logistic regressions were used for associations between socio-economic indicators and lifestyle clusters. Significant differences (*p* < 0.05) are marked as bold.

**Table 4:** Odds ratios and 95% confidence intervals for overweight and obesity depending on lifestyle clusters by sex.

			Overweight		Obesity		Abdominal Obesity	
			OR (95% CI)	*p*	OR (95% CI)	*p*	OR (95% CI)	*p*
Boys	Model 1: Unadjusted	Mixed	1.53 (1.24, 1.90)	**<0.001**	1.50 (1.18, 1.92)	**0.001**	1.27 (1.04, 1.54)	**0.017**
		Unhealthy	1.60 (1.19, 2.14)	**0.002**	2.42 (1.79,3.27)	**<0.001**	1.93 (1.51, 2.48)	**<0.001**
	Model 2: Adjusted	Mixed	1.40 (1.13, 1.74)	**0.002**	1.21 (0.94, 1.56)	0.144	1.19 (0.97, 1.45)	0.092
		Unhealthy	1.31 (0.97, 1.78)	0.077	1.49 (1.08, 2.05)	**0.016**	1.65 (1.27, 2.14)	**<0.001**
	Model 3: Adjusted	Mixed	1.45 (1.17, 1.80)	**<0.001**	1.39 (1.08, 1.79)	**0.011**	1.30 (1.06, 1.58)	**0.011**
		Unhealthy	1.37 (1.01, 1.85)	**0.042**	1.93 (1.41, 2.64)	**<0.001**	1.93 (1.49, 2.50)	**<0.001**
	Model 4: Adjusted	Mixed	1.40 (1.12, 1.74)	**0.003**	1.22 (0.94, 1.58)	0.128	1.21 (0.98, 1.48)	0.069
		Unhealthy	1.25 (0.92, 1.70)	0.149	1.46 (1.06, 2.02)	**0.021**	1.67 (1.29, 2.18)	**<0.001**
Girls	Model 1: Unadjusted	Mixed	1.26 (1.02, 1.56)	**0.033**	1.32 (0.99, 1.77)	**0.059**	1.032 (0.85, 1.25)	0.755
		Unhealthy	1.41 (1.04, 1.92)	**0.027**	2.24 (1.54, 3.24)	**<0.001**	1.35 (1.03, 1.77)	**0.032**
	Model 2: Adjusted	Mixed	1.19 (0.96, 1.48)	0.104	1.15 (0.85, 1.54)	0.362	0.95 (0.77, 1.17)	0.650
		Unhealthy	1.25 (0.94, 1.75)	0.122	1.71 (1.17, 2.51)	**0.006**	1.36 (1.01, 1.83)	**0.039**
	Model 3: Adjusted	Mixed	1.31 (0.95, 1.78)	0.094	1.17 (0.87, 1.58)	0.300	0.99 (0.81, 1.23)	0.968
		Unhealthy	1.24 (1.00, 1.54)	**0.049**	1.66 (1.12, 2.46)	**0.012**	1.42 (1.06, 1.91)	**0.019**
	Model 4: Adjusted	Mixed	1.20 (0.97, 1.49)	0.094	1.08 (0.80, 1.47)	0.602	0.94 (0.76, 1.16)	0.562
		Unhealthy	1.25 (0.91, 1.71)	0.173	1.47 (0.99, 2.19)	0.056	1.28 (0.95, 1.73)	0.106

(a) Reference cluster: Healthy cluster; Reference for weight status: underweight/normal weight. (b) CI: Confidence interval; OR: Odds-ratio. (c) Model 1 is unadjusted; Model 2 is adjusted by age and SES (FAS scale); Model 3 is adjusted by age and food insecurity (HFIAS); Model 4 is adjusted by age, SES (FAS scale), and food insecurity (HFIAS). (d) Multinomial logistic regressions were used for associations between clusters and overweight/obesity, and binary logistic regressions were used for associations between clusters and abdominal obesity. Significant differences (*p* < 0.05) are marked as bold.

## Data Availability

The data presented in this study are available on request from the corresponding author due to ethical restrictions and participant confidentiality. Therefore, data from the ENPIMAD Study are available upon request for researchers who meet the criteria for access to confidential data.

## References

[1] Smith J.D., Fu E., Kobayashi M.A. (2020). Prevention and Management of Childhood Obesity and Its Psychological and Health Comorbidities. Annu. Rev. Clin. Psychol..

[2] Morales Camacho W.J., Molina Díaz J.M., Plata Ortiz S., Plata Ortiz J.E., Morales Camacho M.A., Calderón B.P. (2019). Childhood Obesity: Aetiology, Comorbidities, and Treatment. Diabetes Metab. Res. Rev..

[3] Yan Y., Liu J., Zhao X., Cheng H., Huang G., Mi J. (2019). Abdominal Visceral and Subcutaneous Adipose Tissues in Association with Cardiometabolic Risk in Children and Adolescents: The China Child and Adolescent Cardiovascular Health (CCACH) Study. BMJ Open Diabetes Res. Care.

[4] Viitasalo A., Schnurr T.M., Pitkänen N., Hollensted M., Nielsen T.R.H., Pahkala K., Atalay M., Lind M.V., Heikkinen S., Frithioff-Bøjsøe C. (2019). Abdominal Adiposity and Cardiometabolic Risk Factors in Children and Adolescents: A Mendelian Randomization Analysis. Am. J. Clin. Nutr..

[5] World Health Organization (WHO) Obesity and Overweight. https://www.who.int/news-room/fact-sheets/detail/obesity-and-overweight.

[6] Bravo-Saquicela D.M., Sabag A., Rezende L.F.M., Rey-Lopez J.P. (2022). Has the Prevalence of Childhood Obesity in Spain Plateaued? A Systematic Review and Meta-Analysis. Int. J. Environ. Res. Public Health.

[7] Bertomeu-Gonzalez V., Sanchez-Ferrer F., Quesada J.A., Nso-Roca A.P., Lopez-Pineda A., Ruiz-Nodar J.M. (2024). Prevalence of Childhood Obesity in Spain and Its Relation with Socioeconomic Status and Health Behaviors: Population-Based Cross-Sectional Study. Med. Clin..

[8] Gutiérrez-González E., García-Solano M., Pastor-Barriuso R., Fernández de Larrea-Baz N., Rollán-Gordo A., Peñalver-Argüeso B., Peña-Rey I., Pollán M., Pérez-Gómez B. (2024). A Nation-wide Analysis of Socioeconomic and Geographical Disparities in the Prevalence of Obesity and Excess Weight in Children and Adolescents in Spain: Results from the ENE-COVID Study. Pediatr. Obes..

[9] Poorolajal J., Sahraei F., Mohamdadi Y., Doosti-Irani A., Moradi L. (2020). Behavioral Factors Influencing Childhood Obesity: A Systematic Review and Meta-Analysis. Obes. Res. Clin. Pract..

[10] Liberali R., Del Castanhel F., Kupek E., Assis M.A.A.D. (2021). Latent Class Analysis of Lifestyle Risk Factors and Association with Overweight and/or Obesity in Children and Adolescents: Systematic Review. Child. Obes..

[11] Alosaimi N., Sherar L.B., Griffiths P., Pearson N. (2023). Clustering of Diet, Physical Activity and Sedentary Behaviour and Related Physical and Mental Health Outcomes: A Systematic Review. BMC Public Health.

[12] Seo S.H., Shim Y.S. (2019). Association of Sleep Duration with Obesity and Cardiometabolic Risk Factors in Children and Adolescents: A Population-Based Study. Sci. Rep..

[13] Han S.-H., Yee J.-Y., Pyo J.-S. (2022). Impact of Short Sleep Duration on the Incidence of Obesity and Overweight among Children and Adolescents. Medicina.

[14] Nugent R., Althouse A., Yaqub Y., Nugent K., Raj R. (2014). Modeling the Relation between Obesity and Sleep Parameters in Children Referred for Dietary Weight Reduction Intervention. South. Med. J..

[15] D’Souza N.J., Kuswara K., Zheng M., Leech R., Downing K.L., Lioret S., Campbell K.J., Hesketh K.D. (2020). A Systematic Review of Lifestyle Patterns and Their Association with Adiposity in Children Aged 5–12 Years. Obes. Rev..

[16] Parker K.E., Salmon J., Costigan S.A., Villanueva K., Brown H.L., Timperio A. (2019). Activity-Related Behavior Typologies in Youth: A Systematic Review. Int. J. Behav. Nutr. Phys. Act..

[17] Lee A.M., Scharf R.J., Filipp S.L., Gurka M.J., DeBoer M.D. (2019). Food Insecurity Is Associated with Prediabetes Risk among U.S. Adolescents, NHANES 2003–2014. Metab. Syndr. Relat. Disord..

[18] Au L.E., Zhu S.M., Nhan L.A., Plank K.R., Frongillo E.A., Laraia B.A., Gurzo K., Ritchie L.D. (2019). Household Food Insecurity Is Associated with Higher Adiposity among US Schoolchildren Ages 10–15 Years: The Healthy Communities Study. J. Nutr..

[19] Lee J., Kubik M.Y., Fulkerson J.A. (2019). Diet Quality and Fruit, Vegetable, and Sugar-Sweetened Beverage Consumption by Household Food Insecurity among 8- to 12-Year-Old Children during Summer Months. J. Acad. Nutr. Diet..

[20] Na M., Eagleton S.G., Jomaa L., Lawton K., Savage J.S. (2020). Food Insecurity Is Associated with Suboptimal Sleep Quality, but Not Sleep Duration, among Low-Income Head Start Children of Pre-School Age. Public Health Nutr..

[21] Serra-Majem L., García-Closas R., Ribas L., Pérez-Rodrigo C., Aranceta J. (2001). Food Patterns of Spanish Schoolchildren and Adolescents: The EnKid Study. Public Health Nutr..

[22] Coates J., Swindale A., Bilinsky P. (2007). Household Food Insecurity Access Scale (HFIAS) for Measurement of Food Access: Indicator Guide: Version 3.

[23] Torsheim T., Cavallo F., Levin K.A., Schnohr C., Mazur J., Niclasen B., Currie C. (2016). Psychometric Validation of the Revised Family Affluence Scale: A Latent Variable Approach. Child Indic. Res..

[24] World Health Organization (WHO) (1995). Expert Committee Physical Status: The Use and Interpretation of Anthropometry.

[25] World Health Organization BMI-for-Age (5–19 Years). https://www.who.int/tools/growth-reference-data-for-5to19-years/indicators/bmi-for-age.

[26] World Health Organization Body Mass Index-for-Age (BMI-for-Age). https://www.who.int/toolkits/child-growth-standards/standards/body-mass-index-for-age-bmi-for-age.

[27] Nagy P., Intemann T., Buck C., Pigeot I., Ahrens W., Molnar D. (2016). Erratum: Percentile Reference Values for Anthropometric Body Composition Indices in European Children from the IDEFICS Study. Int. J. Obes..

[28] Browning L.M., Hsieh S.D., Ashwell M. (2010). A Systematic Review of Waist-to-Height Ratio as a Screening Tool for the Prediction of Cardiovascular Disease and Diabetes: 0·5 Could Be a Suitable Global Boundary Value. Nutr. Res. Rev..

[29] Zapico A.G., Aparicio-Ugarriza R., Quesada-González C., Gómez S.F., Wärnberg J., Medrano M., Gusi N., Aznar S., Marín-Cascales E., González-Valeiro M.A. (2023). Lifestyle Behaviors Clusters in a Nationwide Sample of Spanish Children and Adolescents: PASOS Study. Pediatr. Res..

[30] Gebremariam M.K., Totland T.H., Andersen L.F., Bergh I.H., Bjelland M., Grydeland M., Ommundsen Y., Lien N. (2012). Stability and Change in Screen-Based Sedentary Behaviours and Associated Factors among Norwegian Children in the Transition between Childhood and Adolescence. BMC Public Health.

[31] Janssen X., Mann K.D., Basterfield L., Parkinson K.N., Pearce M.S., Reilly J.K., Adamson A.J., Reilly J.J. (2016). Development of Sedentary Behavior across Childhood and Adolescence: Longitudinal Analysis of the Gateshead Millennium Study. Int. J. Behav. Nutr. Phys. Act..

[32] Pearson N., Haycraft E., Johnston J.P., Atkin A.J. (2017). Sedentary Behaviour across the Primary-Secondary School Transition: A Systematic Review. Prev. Med..

[33] Gebremariam M.K., Bergh I.H., Andersen L.F., Ommundsen Y., Totland T.H., Bjelland M., Grydeland M., Lien N. (2013). Are Screen-Based Sedentary Behaviors Longitudinally Associated with Dietary Behaviors and Leisure-Time Physical Activity in the Transition into Adolescence?. Int. J. Behav. Nutr. Phys. Act..

[34] Shqair A.Q., Pauli L.A., Costa V.P.P., Cenci M., Goettems M.L. (2019). Screen Time, Dietary Patterns and Intake of Potentially Cariogenic Food in Children: A Systematic Review. J. Dent..

[35] Rubín L., Gába A., Pelclová J., Štefelová N., Jakubec L., Dygrýn J., Hron K. (2022). Changes in Sedentary Behavior Patterns during the Transition from Childhood to Adolescence and Their Association with Adiposity: A Prospective Study Based on Compositional Data Analysis. Arch. Public Health.

[36] Neufeld L.M., Andrade E.B., Ballonoff Suleiman A., Barker M., Beal T., Blum L.S., Demmler K.M., Dogra S., Hardy-Johnson P., Lahiri A. (2022). Food Choice in Transition: Adolescent Autonomy, Agency, and the Food Environment. Lancet.

[37] Kemp B.J., Parrish A.-M., Cliff D.P. (2020). ‘Social Screens’ and ‘the Mainstream’: Longitudinal Competitors of Non-Organized Physical Activity in the Transition from Childhood to Adolescence. Int. J. Behav. Nutr. Phys. Act..

[38] Pérez-Rodrigo C., Gil Á., González-Gross M., Ortega R., Serra-Majem L., Varela-Moreiras G., Aranceta-Bartrina J. (2015). Clustering of Dietary Patterns, Lifestyles, and Overweight among Spanish Children and Adolescents in the ANIBES Study. Nutrients.

[39] de Mello G.T., Minatto G., Costa R.M., Leech R.M., Cao Y., Lee R.E., Silva K.S. (2024). Clusters of 24-Hour Movement Behavior and Diet and Their Relationship with Health Indicators among Youth: A Systematic Review. BMC Public Health.

[40] Kivimäki M., Vahtera J., Tabák A.G., Halonen J.I., Vineis P., Pentti J., Pahkala K., Rovio S., Viikari J., Kähönen M. (2018). Neighbourhood Socioeconomic Disadvantage, Risk Factors, and Diabetes from Childhood to Middle Age in the Young Finns Study: A Cohort Study. Lancet Public Health.

[41] Sanders T., Feng X., Fahey P.P., Lonsdale C., Astell-Burt T. (2015). The Influence of Neighbourhood Green Space on Children’s Physical Activity and Screen Time: Findings from the Longitudinal Study of Australian Children. Int. J. Behav. Nutr. Phys. Act..

[42] Seabra A., Mendonça D., Maia J., Welk G., Brustad R., Fonseca A.M., Seabra A.F. (2013). Gender, Weight Status and Socioeconomic Differences in Psychosocial Correlates of Physical Activity in Schoolchildren. J. Sci. Med. Sport.

[43] Kral T.V.E., Chittams J., Moore R.H. (2017). Relationship between Food Insecurity, Child Weight Status, and Parent-reported Child Eating and Snacking Behaviors. J. Spec. Pediatr. Nurs..

[44] Ortiz-Marrón H., Ortiz-Pinto M.A., Urtasun Lanza M., Cabañas Pujadas G., Valero Del Pino V., Belmonte Cortés S., Gómez Gascón T., Ordobás Gavín M. (2022). Household Food Insecurity and Its Association with Overweight and Obesity in Children Aged 2 to 14 Years. BMC Public Health.

[45] Yannakoulia M., Lykou A., Kastorini C.M., Saranti Papasaranti E., Petralias A., Veloudaki A., Linos A. (2016). Socio-Economic and Lifestyle Parameters Associated with Diet Quality of Children and Adolescents Using Classification and Regression Tree Analysis: The DIATROFI Study. Public Health Nutr..

[46] Borraccino A., Lemma P., Iannotti R.J., Zambon A., Dalmasso P., Lazzeri G., Giacchi M., Cavallo F. (2009). Socioeconomic Effects on Meeting Physical Activity Guidelines. Med. Sci. Sports Exerc..

[47] Yang-Huang J., van Grieken A., Wang L., Jansen W., Raat H. (2020). Clustering of Sedentary Behaviours, Physical Activity, and Energy-Dense Food Intake in Six-Year-Old Children: Associations with Family Socioeconomic Status. Nutrients.

[48] Leech R.M., McNaughton S.A., Timperio A. (2014). Clustering of Children’s Obesity-Related Behaviours: Associations with Sociodemographic Indicators. Eur. J. Clin. Nutr..

[49] Schröder H., Bawaked R.A., Ribas-Barba L., Izquierdo-Pulido M., Roman-Viñas B., Fíto M., Serra-Majem L. (2017). Cumulative Effect of Obesogenic Behaviours on Adiposity in Spanish Children and Adolescents. Obes. Facts.

[50] Sánchez-Oliva D., Grao-Cruces A., Carbonell-Baeza A., Cabanas-Sánchez V., Veiga O.L., Castro-Piñero J. (2018). Lifestyle Clusters in School-Aged Youth and Longitudinal Associations with Fatness: The UP&DOWN Study. J. Pediatr..

[51] Miguel-Berges M.L., Mouratidou T., Santaliestra-Pasias A., Androutsos O., Iotova V., Galcheva S., De Craemer M., Cardon G., Koletzko B., Kulaga Z. (2023). Longitudinal Associations between Diet Quality, Sedentary Behaviours and Physical Activity and Risk of Overweight and Obesity in Preschool Children: The ToyBox-study. Pediatr. Obes..

[52] Bae J.-H., Choi J.-H. (2021). Gender Disparities in Childhood Obesity and Household Food Insecurity. Nutrition.

[53] Reilly J.J. (2008). Physical Activity, Sedentary Behaviour and Energy Balance in the Preschool Child: Opportunities for Early Obesity Prevention. Proc. Nutr. Soc..

[54] Wang X., Cheng Z. (2020). Cross-Sectional Studies. Chest.

[55] Quinlan C., Rattray B., Pryor D., Northey J.M., Anstey K.J., Butterworth P., Cherbuin N. (2021). The Accuracy of Self-Reported Physical Activity Questionnaires Varies with Sex and Body Mass Index. PLoS ONE.

[56] Ravelli M.N., Schoeller D.A. (2020). Traditional Self-Reported Dietary Instruments Are Prone to Inaccuracies and New Approaches Are Needed. Front. Nutr..

[57] Svedberg P., Nygren J.M., Staland-Nyman C., Nyholm M. (2016). The Validity of Socioeconomic Status Measures among Adolescents Based on Self-Reported Information about Parents Occupations, FAS and Perceived SES; Implication for Health Related Quality of Life Studies. BMC Med. Res. Methodol..

